# Recurrent Cough in a Pediatric Patient From China: A Case Report of Bordetella pertussis Infection With Genomic Insights

**DOI:** 10.7759/cureus.86760

**Published:** 2025-06-25

**Authors:** Mi Yan, Huaide Yang, Lei Xiong, Changjun Tian

**Affiliations:** 1 Infectious Disease, Hunan Normal University Affiliated Zhangjiajie Hospital (Zhangjiajie People’s Hospital), Zhangjiajie, CHN

**Keywords:** antibiotic resistance, bordetella pertussis, genomic sequencing, macrolide resistance, pediatric respiratory infection

## Abstract

We report the case of a six-year-old girl from Zhangjiajie City, Hunan Province, with a history of recurrent cough lasting for a month, characterized by paroxysmal coughing, expectoration, and intermittent fever, with the highest temperature reaching 39℃. Recurrent coughing in pediatric patients presents diagnostic challenges when multiple pathogens and antibiotic resistance are involved. Despite receiving traditional Chinese medicine and antibiotic treatments, the patient’s symptoms persisted. The initial diagnosis failed to identify the cause, although blood tests showed elevated levels of C-reactive protein and serum amyloid A, and chest X-rays indicated signs of bronchopneumonia. The patient tested positive for respiratory syncytial virus and *Haemophilus influenzae*, with a *Mycoplasma pneumoniae* antibody titer of 1:40. Despite treatment with azithromycin, ambroxol, and nebulization, the cough symptoms did not improve, leading to multiple hospital admissions. Subsequent targeted sequencing confirmed infections by *Bordetella pertussis*, human rhinovirus A, adenovirus type 2, and SARS-CoV-2 at different stages. Notably, genomic analysis during the first hospital stay discovered an A2047G mutation in the 23S rRNA gene of *B. pertussis*, indicating resistance to macrolide drugs; however, subsequent tests showed that this mutation had disappeared. This case highlights the complexity of diagnosing and treating persistent cough in pediatric patients and underscores the importance of targeted next-generation sequencing in identifying resistance and co-infections, ultimately enabling more precise and effective treatment strategies for challenging respiratory infections in children.

## Introduction

Recurrent cough represents a significant health challenge among pediatric patients in China, with chronic cough affecting approximately 7.67% (95% confidence interval = 6.24-9.11%) of community-based children nationwide [1]. A recent cross-sectional study in Wuxi, China, found that chronic cough prevalence among children aged 3-18 years reached 15.50%, with affected children demonstrating significantly higher incidences of comorbid conditions, including eczema, wheezing, rhinitis, food allergies, and nasosinusitis [2]. The complexity of diagnosing persistent cough in children has been further complicated by the COVID-19 pandemic, which altered traditional respiratory infection patterns and created diagnostic challenges for clinicians [3].

In China, the resurgence of pertussis has emerged as a critical public health concern. Between 2018 and 2022, among individuals affected by pertussis, infants under one year of age constituted 52.40%, children aged 5-9 years accounted for 13.01%, and children and adults aged 10 years and older accounted for 2.49%. These figures are comparable to those observed in the late 1980s [4].

This concerning trend is not limited to China, as globally, the incidence of *Bordetella pertussis* infection among children is on the rise, particularly among those who have completed primary immunizations [5]. While macrolides such as azithromycin are extensively used in the treatment of pertussis, the therapeutic landscape has become increasingly complex due to the emergence of antibiotic resistance in *B. pertussis *[6]. The A2047G mutation in the 23S rRNA gene identified in this case serves as a clear indication of this developing macrolide resistance [7].

With the growing identification of respiratory pathogens, particularly following the global COVID-19 pandemic, where systematic reviews indicate viral co-infection rates of approximately 3% and early presentation studies report rates as low as 0.5%, the diagnosis and treatment of complex infections pose new challenges [8]. A comprehensive analysis of pediatric respiratory infections indicated that around 9.77% of multiplex polymerase chain reaction tests detected more than one pathogen, emphasizing the complexity of contemporary respiratory infections [9]. The patient was infected with multiple pathogens simultaneously, which not only reflects the necessity to consider the possibility of co-infections when choosing treatment strategies but also highlights the critical need for comprehensive diagnostic approaches in modern pediatric respiratory medicine [10]. Targeted next-generation sequencing (tNGS) has emerged as a revolutionary diagnostic tool that offers superior sensitivity and rapid pathogen identification compared to conventional microbiological methods [11], with demonstrated efficacy in detecting complex co-infections and antimicrobial resistance genes within clinically relevant timeframes.

The impact of bacterial and viral coexistence on the clinical outcomes of patients necessitates advanced diagnostic and therapeutic strategies. This case report aims to demonstrate the diagnostic value of tNGS in identifying complex pathogen interactions and antimicrobial resistance patterns in pediatric respiratory infections. Specifically, we seek to illustrate how genomic surveillance can guide personalized treatment understanding and monitor dynamic resistance profiles and evolving microbial landscapes. Through a detailed analysis of a challenging clinical case involving *B. pertussis* with macrolide resistance (resistance to erythromycin-class antibiotics) and multiple concurrent infections, we aim to provide insights for improving diagnostic protocols and treatment outcomes in pediatric respiratory medicine.

## Case presentation

**Figure 1:**
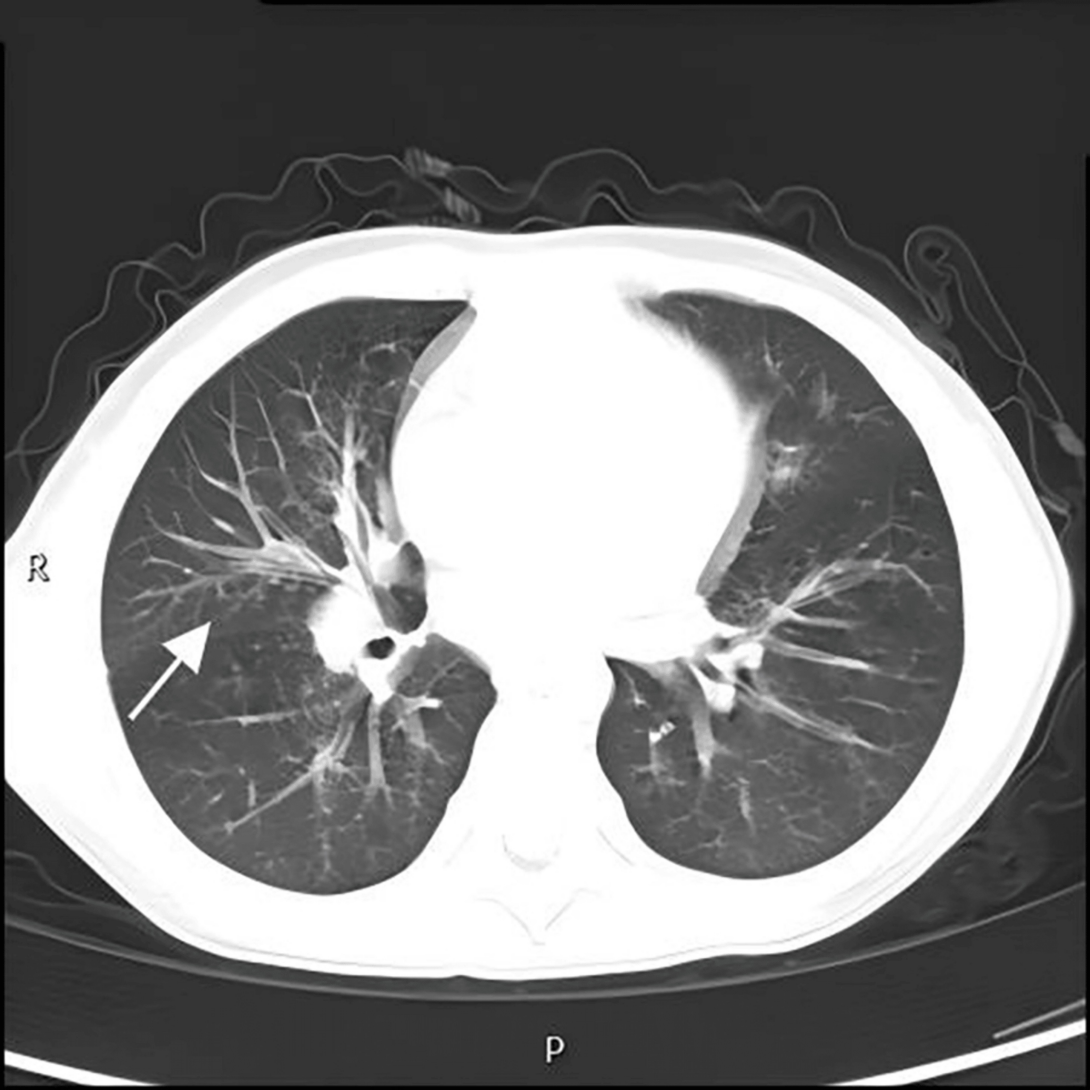
Initial chest radiograph obtained on admission revealing characteristic bronchopneumonia features.

A six-year-old girl from Zhangjiajie, Hunan, China, started coughing one month before being admitted to the hospital. Three days before admission, her cough deteriorated, accompanied by vomiting and intermittent fever, with the maximum body temperature reaching 39℃. Treatment with conventional antibiotics and traditional Chinese medicine failed to relieve the symptoms. The patient was first admitted to our department. Upon admission, her body temperature was 36.5℃, her heart rate was 135 beats per minute (indicating tachycardia), her oxygen saturation was 95% on room air, there was sinus tenderness, and coarse breath sounds were detected in both lungs, suggesting a respiratory infection. Laboratory tests (Table 1) found elevated levels of C-reactive protein (9.54 mg/L) and interleukin-6 (11.10 pg/mL), and positive *Mycoplasma pneumoniae* antibodies (titer 1:40). Chest radiography demonstrated characteristic bronchopneumonia features (Figure 1). Upper respiratory pathogen testing revealed respiratory syncytial virus and *Haemophilus influenzae*, indicating an active immune response and multiple infections. The patient was initially diagnosed with respiratory syncytial virus pneumonia and treated with azithromycin for the infection, ambroxol for expectoration, and a combination of budesonide + ipratropium bromide via nebulization. After six days of treatment, her symptoms improved, and she was discharged with a minor cough. Post-discharge treatment continued with budesonide + ipratropium bromide nebulization and oral administration of a lung-moistening paste to consolidate the therapeutic effect, but her cough symptoms did not alleviate and gradually worsened.

**Table 1:** Summary of laboratory findings and reference ranges across different time points. CRP: C-reactive protein; IL-6: interleukin-6; RSV: respiratory syncytial virus; tNGS: targeted next-generation sequencing; CLSI: Clinical and Laboratory Standards Institute

Date	Test item	Result	Reference range
At admission (initial hospital stay)	CRP	9.54 mg/L	<10 mg/L
	IL-6	11.10 pg/mL	<7 pg/mL
	*Mycoplasma pneumoniae *antibody	1:40 (positive)	<1:40 (negative)
	Chest X-ray	Bronchopneumonia	Normal lung fields
	RSV and *Haemophilus influenzae*	Positive	Negative
Approximately t weeks after admission (second hospital stay)	Tuberculosis test	Negative	Negative
	*Mycoplasma pneumoniae* test	Negative	Negative
	Sputum culture	No growth	No growth (sterile)
	tNGS (upper respiratory)	*Bordetella pertussis* (61,830 seq) RSV-B (14,278 seq) *Haemophilus influenzae* (235 seq)	Negative for pathogens
Approximately 1 month after admission (follow-up test)	tNGS (upper respiratory)	*Bordetella pertussis*, human rhinovirus A, adenovirus type 2, SARS-CoV-2	Negative for pathogens
Antibiotic susceptibility	Erythromycin	Resistant	Susceptible (CLSI guidelines)
	Levofloxacin	Sensitive	Susceptible
	Amoxicillin-clavulanate	Sensitive	Susceptible

The patient was readmitted for further diagnosis and treatment. The admission examination showed severe inflammation and mild liver function impairment. Tests for tuberculosis and *M. pneumoniae* were both negative, excluding the possibility of these two common infections. The sputum culture did not show any abnormalities. Importantly, tNGS of upper respiratory tract pathogens (KingCreate) revealed the presence of *B. pertussis* (61,830 sequences), human respiratory syncytial virus type B (14,278 sequences), and *H. influenzae* (235 sequences), indicating a complex infection situation. It was confirmed as an infection by *B. pertussis*. Moreover, tNGS also identified the A2047G mutation in the 23S rRNA gene, with a frequency of 40%, which suggests that this strain of *B. pertussis* may be resistant to commonly used macrolide antibiotics. During hospitalization, the patient received a seven-day course of azithromycin along with symptomatic supportive treatment, followed by outpatient oral administration of sulfamethoxazole (SMZ, 0.48g, twice a day, for 3-5 days). Due to a lack of improvement, the patient was readmitted.

Subsequent tNGS of upper respiratory pathogens once again identified a complex infection predominantly involving *B. pertussis*. Despite no further detection of resistance genes, the patient was switched to treatment with amoxicillin-clavulanate potassium for the bacterial infection, accompanied by six days of supportive therapy. Following this, the patient’s symptoms improved, and the patient was then discharged.

**Figure 2:**
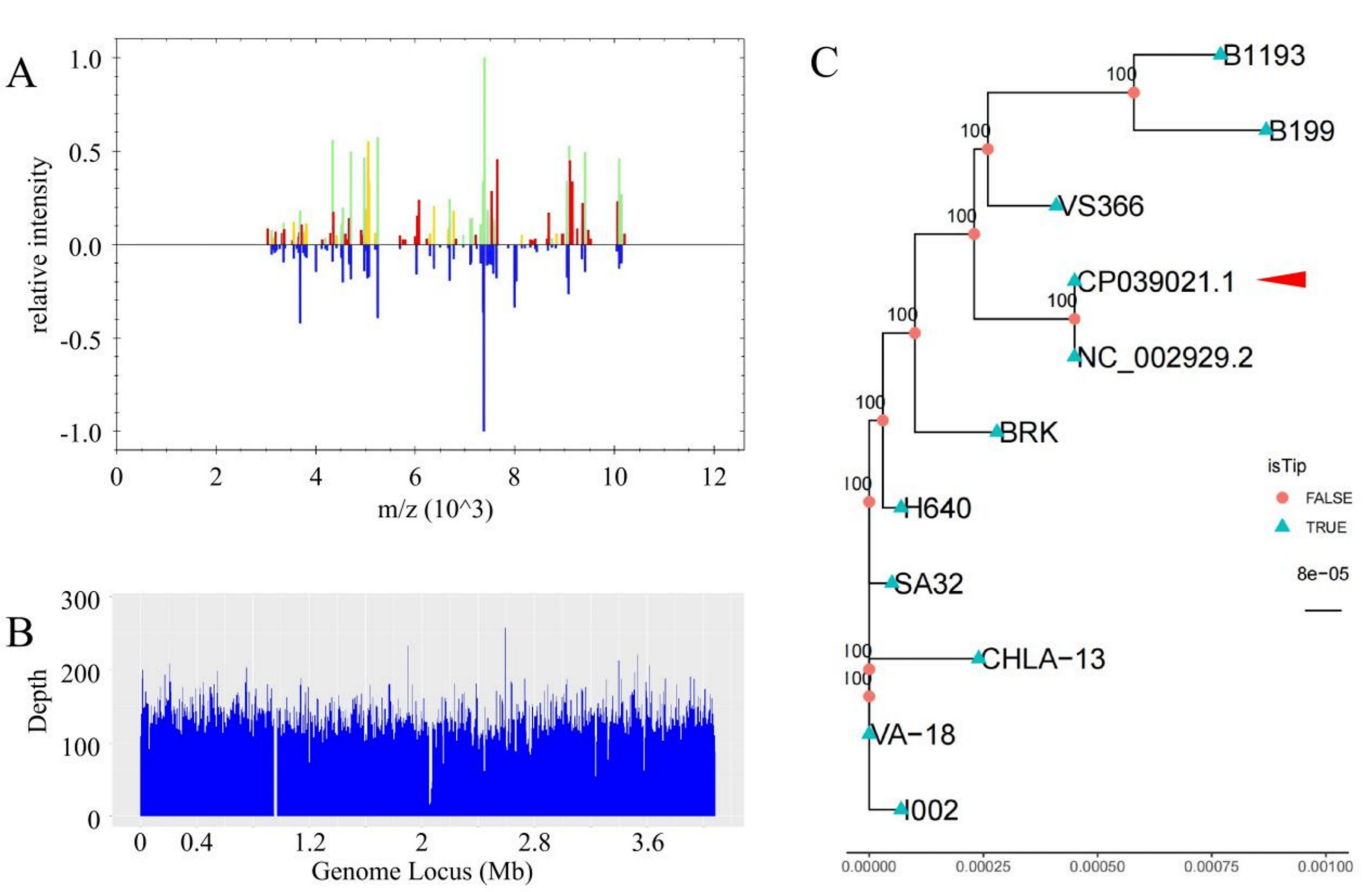
(A) Mass spectrometry identification. Mass spectrometry was utilized to identify proteins from Bordetella pertussis, aiding in the understanding of the bacterial protein profile and potential virulence factors. (B) Genome sequencing coverage showing the relationship between the genome locus and depth of the sequencing coverage. (C) Phylogenetic analysis of B. pertussis strains. A comprehensive analysis was conducted using sequences from publicly available B. pertussis strains, compared against a selected reference genome for the analysis. The genomic sequence alignment with the reference genome revealed a high similarity rate of 97.13%. Distinct annotations within this reconstruction clearly identify terminal nodes (isTip = True) and internal nodes (isTip = False), underscoring the detailed evolutionary connections among the strains relative to the reference genome.

Subsequent efforts were first directed toward isolating and culturing the pathogen, followed by accurate identification of the pathogen as *B. pertussis* using mass spectrometry identification technology (BRUKER, MALDI Biotyper), with Figure 2A displaying the mass spectrometry results. Although the patient had completed the standard childhood vaccination schedule according to Chinese immunization guidelines, including four doses of diphtheria-tetanus-pertussis vaccine administered at 3, 4, 5, and 18 months of age. Through genome sequencing, using specifically published *B. pertussis* strains, and with NC_002929.2 as the reference genome (alignment rate of 97.13%), a phylogenetic analysis (evolutionary relationship study) was constructed (Figures 2B, 2C). On this basis, by analyzing the genomic sequence data in VCF format, we discovered several mutations within the 23S rRNA gene, among which the A2047G mutation (located on the X68323.1 reference sequence, showing a transition from A to G, with a frequency of 40%) might indicate macrolide resistance. This finding provides additional molecular evidence for our phylogenetic tree, showcasing the possible resistance differences among strains.

To determine the most effective antibiotic treatment regimen, we performed antibiotic susceptibility testing on the isolated *B. pertussis*. The results showed the pathogen’s susceptibility to various antibiotics. These results are detailed in Table 1.

## Discussion

Treatment strategy and antibiotic management

The treatment first employed azithromycin, guided by early genomic testing to effectively address the initial pathogen. As new diagnostic insights and responses emerged, the regimen shifted to amoxicillin-clavulanate, targeting *B. pertussis* strains without macrolide resistance and broadening the fight against other infections. This alternative approach has demonstrated effectiveness against *B. pertussis* in pediatric populations and is recommended as treatment for macrolide-resistant strains [12,13].

Supportive treatments such as expectorants, nebulization therapy, cough suppressants, and bronchodilators played a key role in symptom relief and respiratory improvement. These supportive measures are well-established components of pertussis management, with cough suppressants and bronchodilators showing efficacy in reducing symptom severity and improving respiratory function [14,15]. Post-discharge, SMZ was used for its wide antibacterial coverage, aligning with resistance monitoring to ensure the treatment approach’s effectiveness. Sulfamethoxazole-trimethoprim has been demonstrated as an effective alternative treatment for pertussis, particularly in cases of macrolide resistance [7].

Genomic analysis and resistance mechanisms

This case report presents an in-depth genomic analysis of the *B. pertussis* infection and the application of antibiotic resistance testing. Initially, we discovered the A2047G mutation in the 23S rRNA gene through genomic sequencing, suggesting a potential resistance to macrolide antibiotics. This mutation has been extensively documented in earlier studies as the primary mechanism of macrolide resistance in *B. pertussis *[16]. Interestingly, the mutation disappeared in subsequent tests, revealing the complexity of bacterial resistance. The disappearance of the A2047G mutation in subsequent tests can be explained by the fitness cost associated with antibiotic resistance mutations and the dynamic nature of bacterial populations [17]. While methodological variance in sampling or sequencing sensitivity cannot be entirely excluded, the temporal pattern observed suggests genuine genomic plasticity rather than technical artifacts, as confirmed by consistent sequencing protocols and quality controls throughout the study period.

Through the construction of a phylogenetic tree incorporating data on the A2047G mutation in the 23S rRNA gene and the novel fhaB3 allele, we deepened our understanding of the genetic relationships between the erythromycin-resistant *B. pertussis* strain isolated in this case and three independent erythromycin-resistant lineages identified across China [18]. Mass spectrometry, in concert with phylogenetic analysis, proved indispensable in delineating the evolutionary trajectories of the pathogen, reflecting the impact of vaccination and antibiotic use on the genetic diversity of *B. pertussis*.

Clinical implications and public health significance

Antibiotic susceptibility testing showed high sensitivity to levofloxacin and low sensitivity to erythromycin, indicating the necessity to adjust treatment plans based on actual drug sensitivity results in clinical practice. This finding aids in optimizing personalized treatment strategies. Notably, *B. pertussis* isolates may display varying degrees of macrolide resistance, further supporting the importance of antimicrobial susceptibility testing in guiding treatment decisions [19]. Specifically, the high prevalence of erythromycin-resistant *B. pertussis* in China (97.6%) is associated with the A2047G mutation [20]. This resistance pattern provides important guidance for clinical medication, emphasizing the need for greater reliance on drug sensitivity test results to select effective antibiotics when treating pertussis [18].

This case report not only has implications for improving the treatment success rate of individual cases but also provides important references for public health monitoring and addressing the challenge of antibiotic resistance [21]. Recent literature also supports our case findings, including the association between severe clinical manifestations and co-infections or macrolide treatment failure [22], the occurrence of pertussis resurgence in fully vaccinated children [23], and the critical role of next-generation sequencing in diagnosis [24]. Together with this case, they suggest that the application of tNGS, routine susceptibility testing for macrolide resistance, and establishing a follow-up protocol incorporating repeated molecular testing can prevent diagnostic delays in similar cases and optimize treatment outcomes.

## Conclusions

The emergence of complex co-infections involving *B. pertussis*, respiratory syncytial virus, human rhinovirus A, adenovirus type 2, and SARS-CoV-2 underscores the critical need for advanced diagnostic modalities, such as tNGS, to delineate pathogen interactions and optimize therapeutic strategies. However, it is important to acknowledge that tNGS implementation faces significant limitations, particularly regarding cost-effectiveness and accessibility in resource-limited healthcare settings, which may restrict its widespread adoption for routine pediatric respiratory infection diagnosis. Genomic surveillance revealed transient macrolide resistance via the A2047G mutation in *B. pertussis* 23S rRNA, highlighting the dynamic nature of antimicrobial resistance profiles that necessitate real-time pathogen characterization during treatment courses. This case emphasizes that comprehensive diagnostic approaches integrating microbial identification, antibiotic susceptibility testing, and resistance gene monitoring are indispensable for guiding antibiotic stewardship and improving clinical outcomes in pediatric respiratory infections.
